# Rumor as ethical vernacular: Ebola and the womb in eastern Congo

**DOI:** 10.1111/maq.70015

**Published:** 2025-07-22

**Authors:** Myfanwy James

**Affiliations:** ^1^ Department of International Development London School of Economics and Political Science London UK

**Keywords:** DRC, Ebola, ethics, pregnancy, rumor, sterilization

## Abstract

The position of pregnant women in clinical research remains a topic of international ethical debate. Yet, the reflections of actual and potential trial participants, including pregnant women themselves, often remain absent. Following a policy reversal in 2019, pregnant women were eligible to participate in a second Ebola vaccine trial during an epidemic in the eastern Democratic Republic of the Congo (DRC). This article follows how this decision was perceived in Goma, a city in the DRC, the meanings and functions of the rumors that emerged about reproductive health, and how these rumors influenced pregnant women's experience of the trial. I argue that the womb became a site to discuss broader biopolitical anxieties about collective survival, but that rumors also became a vehicle for ethical debate amid uncertainty. Ethical debates about medical research continue locally through other ethical vernaculars‐ like rumors‐ and center on contested ideas of acceptable risk, shaped by collective historical experiences.

## INTRODUCTION

The Ebola vaccine clinic was on the side of a busy road in Goma, the capital of North Kivu in the eastern Democratic Republic of the Congo (DRC). The clinic was busy that morning; I joined the queue of people waiting at the entrance for their temperature to be taken. As I entered, one of the nurses asked whether we could discuss something in his office. Amid posters about Ebola now fraying behind COVID‐19 posters, the nurse turned to the subject at hand: pregnant women in the Ebola vaccine trial. He was concerned about the rumors circulating in the neighborhood about the sterilizing impacts of the vaccine and the harmful effects on pregnant women.

There have been cyclical Ebola epidemics in the DRC since the discovery of the virus in the country in 1976. Different vaccine trials have aimed to reduce the spread of the virus and develop new treatments. I was in Goma during DRC's 10th Ebola epidemic (2018–2020), the deadliest the country has ever experienced, when two experimental vaccines were deployed. The first, manufactured by Merck, was administered in a ring vaccination to protect healthcare workers and contacts of Ebola cases. However, as cases continued to rise, another vaccine was introduced to expand coverage. Subsequently, the Janssen trial took place in Goma between November 2019 and February 2021, conducted by the Ministry of Health and a consortium of Congolese and international institutions (James & Lees, 2022).

The position of pregnant women remains a controversial component of clinical trials, and the criteria for deciding their inclusion or exclusion have varied over time and across different trials. Pregnant women were initially excluded from Ebola vaccine trials. However, given the severity of the outbreak, 10 months into the 10th DRC epidemic, there was a reversal in policy to include pregnant and breastfeeding women at the end of the Merck vaccine rollout (Schwartz, 2020). Subsequently, from the start of the Janssen vaccine trial, all adults and children aged 1 year or above, including pregnant and breastfeeding women, were invited to participate.

There were many concerns about the second Ebola vaccine in eastern DRC: that it is a business opportunity for elite enrichment, a covert means to test a COVID‐19 vaccine, or a tool for powerful outsiders to exterminate the population (James & Lees, 2022). Rumors overlapped and drew on experiences of colonial extraction, Rwandan incursions, and protracted insecurity (James et al., 2021). One recurrent theme was reproductive health. Broader biopolitical concerns about collective survival were often expressed through the idiom of sterilization, with fears that the vaccine would target reproductive health in the region as a covert form of gradual extermination. The position of pregnant women in the trial, therefore, became particularly sensitive—a touchpaper for setting off anxieties about the future, where the effect of the vaccine on reproductive health might be observed in real time.

In this article, I examine how the decision to include pregnant women in the Janssen Ebola vaccine trial was perceived in Goma, the meanings and functions of the rumors about reproductive health that emerged, and how these rumors influenced pregnant women's experience of the trial. I make three points. First, the position of pregnant women in global vaccine research in eastern DRC became a forum to discuss broader societal concerns about extermination in the context of fractious state‐society relations, regional conflict, and global asymmetries of power. Rumors are not simply “misinformation” caused by a knowledge deficit, but become vehicles for articulating political anxieties in moments of upheaval (Geissler & Pool, 2006; Masquelier, 2002; Taussig, 1987). Rumors about vaccines and clinical trials, for example, describe the histories of “extractions and invasions” in peoples’ daily lives (White, 2000, 5), situated within imperial and postcolonial histories of medical violence as well as contemporary geopolitical inequities (Fairhead et al., 2006; Geissler, 2005; James et al., 2021; Tilley, 2011). Recurring rumors about sterilization plots express concerns about collective survival (Geissler & Pool, 2006; Renne, 2006): the idiom of threats to local reproductive capacity “reveals a feminine side to local‐global relations, a politics of the womb” (Feldman‐Savelsberg et al., 2000, 159).

However, defining rumors as simply symbolic narratives risks presenting “what appears to fall outside the conventional terms of practical reason as simply a strategic form of symbolic explanation” (Butt, 2005, 417). It is important to also consider why people share rumors that they do not believe, how rumors affect the lives of those they discuss, and why particular rumors acquire such power (Butt, 2005; Geissler & Pool, 2006; Samuels, 2015). In the second section, I show how rumors about reproductive health influenced pregnant women in the trial. Many tried to navigate uncertainty through pragmatism: simultaneously hopeful and anxious in their decision to try experimental therapeutics. Rumors are, therefore, not only reflections of societal anxieties, but can create anxieties, including for people who approach clinical trials as a source of hope in uncertain times.

Finally, I shift the focus from why people might believe rumors to why people *do* rumors. Rumors are not just reflections of political anxieties but can become tools that people share and play with, even if they do not necessarily believe these rumors to be “true.” Rumors become ethical vernaculars: vehicles for ethical debate, or a way of grappling with the contentious ethics of clinical research in an epidemic. Central to ethical discussions about clinical research are contested notions of “acceptable risk” (Graham, 2019). These assessments of risk are not “objective” but rather moral calculations that cannot be separated from historical experiences. The policy reversal to include pregnant women in Ebola vaccine trials did not end debate or uncertainty. Instead, over time, the experiences of pregnant women became key to the production of two forms of “evidence” about the trial: translated into novel data points for the trial team to assess “safety outcomes,” whilst circulating as narrative fragments of “evidence” in Goma. “Doing” rumors, then, did not constitute spreading disinformation, but rather a means to make sense of the potential effects of the vaccine, the “acceptability” of risk, and therefore to grapple with the ethics. Ultimately, this illustrates how ethical debates about medical research are not “finished” in international public health institutions but continue at a local level through other vernaculars (such as rumors) used to express ambivalence and search for answers amid uncertainty.

My analysis is informed by ethnographic research on the politics of international humanitarian intervention in North Kivu since 2017. I was in Goma when the 10th Ebola outbreak began, and as the global epidemic response arrived, I became interested in the vaccine trials undertaken in the region. In 2020, I returned for 8 months to conduct research on experiences and perceptions of the Janssen vaccine trial. This involved observation around the city and in vaccination clinics, as well as interviews with trial participants, concerned citizens, and political authorities about their perceptions of Ebola, the response, and clinical research. This article, in particular, draws on 46 interviews^1^ and five focus groups with trial participants who were approached at the clinics, as well as eight interviews and three focus groups with political authorities and civil society activists who did not participate in the trial. This constituted a “sub‐study” of the trial protocol which was facilitated by the biomedical trial team and enabled my access to vaccine sites. However, I maintained a degree of autonomy: the research was kept distinct from the running of the trial, and systems were put in place to maintain confidentiality. Ultimately, my role became viewed by participants and the trial team alike as an “evaluation,” providing a potential forum for critique.

## PREGNANCY, RISK, AND ETHICS IN CLINICAL TRIALS

The position of pregnant and breastfeeding women in clinical trials remains a subject of international ethical debate. Historically, pregnant women have been excluded from clinical studies due to concerns about fetal risk (Blehar et al., 2013). In 1977, the US Food and Drug Administration (FDA) called for the exclusion of women “with child‐bearing potential” in clinical studies, after thalidomide had been linked to thousands of serious adverse complications (Graham, 2019). Critics disputed this exclusion, highlighting that the thalidomide side effects were the result of pregnant women's exclusion from clinical studies, meaning that they are taking drugs without any safety data on pregnancy (van der Graaf et al., 2019). This led to calls to include more pregnant women in clinical research to avoid approving treatments without sufficient safety data (Blehar et al., 2013).

There was a shift in policy in the 1990s, with growing calls from advocacy groups for clinical research that addresses the needs of pregnant women. The Council for International Organizations of Medical Sciences concluded that the exclusion of pregnant women was “unjust,” and the principle of “presumed eligibility” was adopted by the FDA and European Medicines Agency (van der Graaf et al., 2019). However, in practice, most vaccine trials against infectious diseases continued to exclude pregnant women (Graham, 2019), and the health needs of pregnant women remain under‐researched (van der Graaf et al., 2019). As a result, only 5% of new pharmaceuticals with FDA approval between 2003 and 2012 included data from pregnant women (Heyrana et al., 2018).

The debate about whether to include pregnant women in clinical research has become particularly fractious in epidemics, with debate as to how an “exceptional” context changes the risk‐benefit analysis (Graham, 2019; Schwartz, 2020). Even though pregnant women experience disproportionately high morbidity and mortality, they are often excluded from efforts to develop vaccines (Schwartz & Graham, 2020). This debate became particularly salient during the development of Ebola vaccines in deadly epidemics when pregnant women were initially excluded. Central to ethical debates about clinical research are contested ideas of “acceptable risk”: competing understandings of risk and benefit are an inherently ethical conversation (Graham, 2019; Heyrana et al., 2018). As Graham (2019, 208) argues, in the debate over the inclusion of pregnant women in vaccine research, “myriad risk epistemologies are at work, informed by profession, precedent, geography and social context.” Dominant understandings of pregnant women in the biomedical sphere as “vulnerable,” as well as ambiguity around the principle of “acceptable risk,” are key to their exclusion (Heyrana et al., 2018). In contrast, critics of the decision to exclude pregnant women from Ebola vaccine research highlighted that given the high maternal and fetal mortality rate of Ebola, the potential benefit of inclusion outweigh the risk (Graham, 2019).

The ethics of pregnancy in clinical trials is thus a longstanding debate among global health specialists; however, potential “research participants” themselves are rarely involved in these discussions (Geissler & Pool, 2006), and the assessments of risk among pregnant women themselves remain largely absent (Graham, 2019). I shift the focus to examine how the policy reversal to include pregnant women in an Ebola vaccine trial was perceived among actual and potential trial participants, including pregnant women. This is important because ethical debates are not “finished” after the decisions made in international ethics review boards; instead, these debates continue where trials are implemented, as people grapple with the uncertainty.

Rather than an objective reality, ideas of “acceptable risk” in ethical debates about clinical research cannot be separated from shared historical experiences (Graham, 2019). Contemporary debates about medical research in Africa are sites in which people articulate broader concerns about social justice, opaque workings of power, and historical wrongdoing (Fairhead et al., 2006; Tilley, 2020). While bioethical frameworks center on standardized protocols, ethnographic accounts illustrate the importance of placing bioethics in political context, exposing the limits to bioethical discourses amid profound inequalities (Fairhead et al., 2006), and considering the “imperial origins as well as asymmetrical topography of power and resources” inherent in medical research (Geissler 2011, 1).

Rumors, then, are not just reflections of political anxieties, but are also part of ongoing ethical debates about global epidemic research. As Geissler and Pool highlight (2006, 975), it is critical to “recognise study subjects as interlocutors in ongoing global ethics debates, not as mere objects of ethical responsibility.” Too often, ethical debates about medical research are understood to take place in the international ethics boards, whilst controversy among potential or actual trial participants is framed as “misconceptions” to be countered by “community engagement.” This obscures how ethical debates continue at a local level through other idioms—including rumors—that people use to “debate current events and concerns” (Geissler & Pool, 2006, 980). Beyond questions of “belief,” rumors provide people a means to articulate ambivalence related to the benefits and risks of participation in clinical research, and to muddle through the ethics of medical research in their wider political context.

## EBOLA, VIOLENCE, AND VACCINES

Between July 2018 and June 2020, the DRC's 10th and largest Ebola epidemic unfolded in Ituri, North Kivu, and South Kivu provinces, killing 2280 people. The response was led by the Congolese Ministry of Health, with support from the WHO. The epidemic began in the Grand Nord of North Kivu, which has a long history of violent conflict and rebellion from the central state. This was the first Ebola epidemic in eastern DRC and an active conflict, and attacks against health personnel complicated the response (Sweet & Kasali, 2024).

Distrust of the epidemic response was rooted in decades of conflict and government neglect, as well as frustration toward protracted international intervention. North Kivu has been at the center of violent conflict in the region for the last 30 years. In 1994, conflict became regionalized when Rwandan refugees arrived, and militias responsible for the genocide remobilized in camps. In 1996, the Rwandan army and *L'Alliance des Forces Democratiques pour la Liberation du Congo‐Zaire* (AFDL) invaded, dismantled the camps, and overthrew President Mobutu. However, when the AFDL fell out with their Rwandan backers, another Rwandan‐backed rebel group invaded in 1998. This sparked the Second Congo War, which involved eight countries and more than 25 armed groups. During this time, North Kivu was governed by Rwandan and Ugandan‐backed rebel groups.

The war officially ended in 2003, but violence has continued, and over 100 armed groups now operate in the region. In the Petit Nord, there have been further Rwandan‐backed rebellions led by Congolese Rwandophones, such as the *Congrès National pour la Defense du Peuple* (CNDP) from 2005 to 2009, and the *Mouvemente du 23‐Mars* (M23) from 2012 to 2013, and since 2021. “Mai‐Mai” militia have mobilized in “self‐defence,” claiming to defend those “born from the soil” from Rwandan invaders. Decades of conflict have produced a new class of actors invested in maintaining the status quo (Stearns, 2022). The capital of North Kivu, Goma, is known as the headquarters of peace and war: *siege de la rebellion* during the first and second Congo wars, and a regional hub for international humanitarian agencies and the UN peacekeeping mission, which arrived in the 1990s and has stayed ever since. This protracted international presence has reshaped Goma's political economy, creating a real estate boom, dollarization of the economy, and growing urban inequality (Büscher & Vlassenroot, 2010). The city has grown from 150,000 inhabitants to 2 million and has become a site for political mobilizations in response to insecurity and electoral politics.

In the Grand Nord, since 2013, there have been a series of massacres of civilians committed by an opaque network of armed actors, and the population has grown frustrated at the inability of the government or UN peacekeepers to provide security (Bisoka et al., 2021; Sweet & Kasali, 2024). It was in this context that the first case of Ebola emerged, just thirty kilometers from the site of a recent massacre. People in North Kivu watched as international and national institutions mobilized vast resources to respond to the Ebola outbreak, in stark contrast to the international indifference and governmental inaction in the face of civilian massacres, protracted insecurity, and other health concerns (Sweet & Kasali, 2024). In Goma, there was outrage and solidarity, particularly among civil society movements and people with family connections in the Grand Nord.

Ultimately, many in the region concluded the Ebola response aimed to benefit foreign intervenors or national elites, rather than the population. Staff from abroad and Kinshasa were paid substantially more than locals (Bisoka et al., 2021). Meanwhile, the injection of funds created a clientelist system centered around rental contracts at inflated prices, job kickback schemes, and political side payments, as elites harnessed the Ebola economy for electoral and financial gain (Villa, 2023). This was coined “Ebola business,” a popular critique of the epidemic political economy, which led many to conclude that responders had incentives to prolong the outbreak, or even invent Ebola altogether (Bisoka et al., 2021). The Ebola response collaborated with state security forces, who were locally suspected of involvement in massacres (Sweet & Kasali, 2024), and in December 2018, elections were cancelled in Ebola‐affected regions. This led to protests as many in the region concluded that Ebola was a tool to suppress the opposition stronghold, or a weapon to eliminate the population. In this context, attacks on the Ebola response became a means of expressing political discontent (Bisoka et al., 2021).

Goma did not see many Ebola cases, but was established as the coordination hub for the response, with a new laboratory constructed in the city. The first Ebola case in Goma was confirmed in July 2019. Fearing a rapid spread in the cross‐border transit hub of a million people, the WHO announced a public health emergency of international concern. Yet only four cases were ever confirmed in the city. *Gomatraciens* (residents of Goma) with experience in the epicenter of the epidemic were anxious that Ebola was looming on the horizon. Others, however, were suspicious of a largely elusive threat that had mobilized so many resources. As one trial participant told me, “Ebola is just something we hear about on the radio.” St Thomas became a recurrent motif in conversations about Ebola, referring to the idea that seeing with your own eyes is necessary to believing. The idea of Ebola as “business” resonated in the city because it drew on existing idioms that questioned protracted, external intervention: depicting intervenors as “*pompier pyromanes”* (arsonist firefighters) invested in prolonging crisis, to keep themselves in business. This became a commentary on the contradictions of 30 years of “emergency” intervention, and the unequal political economy it had built.

Two vaccines were part of the epidemic response. The Merck vaccine was first deployed in the Grand Nord in a ring vaccination to stop transmission. Initially, pregnant and breastfeeding women were not eligible. This decision was criticized given the fatal outcomes for fetuses and newborns of Ebola‐infected women (Schwartz et al., 2019). As the epidemic continued, pregnant women in the Grand Nord felt as though they were being “sent to their death.” (Schwartz, 2020). In June 2019, this policy was reversed given the severity of the outbreak: pregnant women beyond their first trimester and breastfeeding women could receive the Merck vaccine if they were identified as case contacts (Schwartz, 2020).

In May 2019, amid concerns of Merck shortages, the WHO recommended introducing a second vaccine to help stop transmission. The proposed use of a second vaccine sparked international debate about the ethics of further vaccine research in epidemic contexts when a vaccine was already approved (Monrad, 2020). The start of the trial was delayed amid political controversy, as the Minister of Health voiced concerns about using the population as “guineapigs” (James & Lees, 2022). After the Minister's high‐profile recognition, civil society groups in Goma questioned the ethics behind testing another vaccine and accused the government of prioritizing pharmaceutical business interests over citizens’ well‐being. As one activist in Goma summarized, he was concerned about why the DRC was accepting further experimentation in poorer neighborhoods of Goma, “especially when a vaccine already exists!” The second vaccine was seen as further proof that Ebola was a “business” to enrich powerful outsiders and co‐opted elites.

Nonetheless, in November 2019, the Janssen trial began in Goma, as a Phase 3 non‐randomized, single‐arm evaluation of the effectiveness, safety, and immunogenicity of a heterologous two‐dose vaccine for the prevention of Ebola in adults and children aged 1 year or above. Unlike the ring vaccination, 20,000 people were eligible to volunteer to participate, including pregnant and breastfeeding women. Whilst the epicenter of the epidemic unfolded in the Grand Nord, the aim here was to instead create a protective “curtain” to prevent the virus from spreading further into the province, while the vaccine was also administered by the Rwandan government across the border. The trial took place at six vaccination clinics in two health areas in Goma—Majengo and Kahembe—selected based on their potential risk of Ebola transmission. Majengo is a “northern port” of the city with a large Nande population with family, trade, and political links to the Grand Nord, while Kahembe is a trading center on the Rwandan border. Plans for vaccination in the Grand Nord were cancelled when the outbreak ended. The trial provided participants with free medical care for 1 month following vaccination, and pregnant women with free care until delivery. 1221 pregnant women participated in the trial, and no “serious adverse events” in pregnant women were considered related to vaccination (Kavunga‐Membo et al., 2024).

## EXTERMINATION, STERILIZATION, AND THE WOMB

The inclusion of pregnant women in the trial became sensitive amid broader societal concerns about extermination and sterilization. Whilst the southern neighborhoods of Goma line the shore of *Lac Kivu* with fortified aid compounds and restaurants, the trial took place in poorer *quartiers populaires*. Majengo, on the northern periphery, has a reputation for being a *“coin chaud”*: a site of contentious politics with frequent political violence and protests. Kahembe is a highly concentrated *quartier populaire* located more centrally, straddling the no‐mans‐land along the Rwandan border. As the clinics opened in these busy neighborhoods, lively conversations began about the potential risks and benefits for pregnant women.

In a church opposite one of the clinics in Kahembe, some trial participants described the inclusion of pregnant women as a novel opportunity, given that they had not been eligible for the first vaccine. Participants highlighted the trial's provision of free medical care for 1 month and free care for pregnant women until delivery as the key benefit of participation, and some participants even recommended that pregnant women volunteer for this reason. Other *Gomatraciens*, however, were concerned that this free healthcare might persuade women, who might otherwise have struggled to pay for healthcare during pregnancy, to participate in the trial despite the risks: “They [the trial] guarantee free healthcare, and they pay the fees for giving birth, so people who do not have money went to be vaccinated, but it wasn't of their own choosing,” a man living in Majengo concluded.

Many in Goma were unsure why the eligibility requirements had changed for the second Ebola vaccine. Soon, rumors circulated that the vaccine would exterminate or sterilize the population, and put those who were already pregnant in danger. In a heated discussion with civil society representatives, one neighborhood leader summarized the mood: “People say that all the girls who receive the vaccine will not have children, that all the pregnant women who receive the vaccine die in hospital.” Those who had already volunteered for the vaccine became increasingly concerned by these rumors. “People say that women will no longer be fertile, they will become sterile, and for men, they won't be able to live more than 50 years. This is the rumor we hear. I suppose we will just have to see,” explained a woman in Majengo. Another woman who had participated in the trial added: “People say that those who take the vaccine, it will make us infertile, we will not be able to have children!” Even trial participants who were not concerned for their own safety were troubled by the rumors: at the end of one interview, a participant concluded, “My worry is just to see how pregnant women do in the trial.”

Since the colonial period, vaccination campaigns in Africa have been suspected of reducing the fertility of young women (Geissler & Pool, 2006; White, 2000). Rumors about sterilization plots articulate concerns about “reproductive bodies, collective survival and global asymmetries of power” (Kaler, 2009, 1711; Renne, 2006; Yahya, 2007). As Feldman‐Savelsberg et al. (2000, 159) explain, rumors about reproductive capacity are part of a “politics of the womb”: the womb becomes a site for struggles over power and control, as local responses to global and national projects play out through the “idiom” of sterilization. To better understand the politics of the womb, such rumors need to be read in context. During the 1990s in Cameroon, for instance, Feldman‐Savelsberg et al. (2000, 159) trace the story of a rumor that the central government were administering a tetanus vaccine to sterilize girls in the west and northwest provinces. This region has a long history of political struggle with the central state, and a new constitution had provoked growing regionalism and crackdowns on opposition. The central government had also just launched a campaign promoting contraceptives at the same time as the vaccination campaign. The sterilization rumor, therefore, drew on historical experiences and contemporary political anxieties about survival and central domination.

In Goma, the position of pregnant women in the Ebola vaccine trial became embroiled in broader concerns about collective survival in the face of violence and uncertainty. As one *chef de quartier* (neighborhood chief) concluded: “The vaccine arrived in a difficult context.” Multiple rumors about sterilization described different global and national extermination schemes that threatened North Kivu, drawing on recent histories of contentious state‐society relations, as well as foreign domination and resource extraction. In one version of these rumors in Goma, Western states were using the vaccine to reduce the Congolese population, so that Europe or the USA could gain control of the mineral rich region. “Whites want to reduce the number of Africans…they want to bring the vaccines so everyone will be sterile,” one man from Majengo summarized. A trial participant added, “I have worries and fears like most people. Everyone says that the aim of the vaccination was to eliminate us from the earth, reduce the global population.” The intention, another trial participant explained, was to sterilize the population, so that “in the future, we won't have anyone to defend our land.” These anxieties intensified as the COVID‐19 pandemic began, generating global ethical debate about vaccine trials in Africa, as well as rumors of sterilization (Tilley, 2020). A rumor circulated in Goma that Europeans had secretly replaced the Ebola vaccine with a sterilizing COVID‐19 vaccine (James & Lees, 2022).

This focus on Western plots reflected concerns about the interference of powerful outsiders in Africa, drawing on histories of violent domination and resource extraction. Rumors draw on collective memories of unregulated and coercive colonial medical campaigns in the region when Africa was viewed as a “living laboratory” by European regimes (Lachenal, 2017; Tilley, 2011), as well as more recent cases of unethical drug testing by pharmaceutical companies in Africa (Graboyes, 2015). Biomedicine was introduced as a tool of early colonial rule in the DRC: the Belgian administration suppressed traditional healers and directed the population to Belgian‐run hospitals (Hunt, 2015). Early encounters with colonial rule involved violent medical campaigns and ineffective treatments for sleeping sickness (Lyons, 1992). Mass trials of Lomidine were conducted in the Belgian Congo, including in Kivu, often without consent and sometimes by force, but the drug was both ineffective and harmful (Lachenal, 2017, 61).

The womb has also long been a site of power struggle and violence in the DRC. Sexual violence was central to colonial power in the Congo Free State: sexual abuse and the stealing of women occurred alongside the more systematic hostage‐taking to enforce rubber collection (Mertens, 2023). Sexual violence, including by external intervenors and mercenaries, remains part of a continuum of systematic violence that is deeply rooted in the historical exploitation of the country (Mertens, 2023). As Nancy Hunt (1999, 8) describes, childbirth then became an “arena of colonial bargaining and strife,” as missionaries, colonial and postcolonial officials attempted to medicalize birthing practices. Today, maternity clinics remain “sites of debate and negotiation” (Hunt, 1999, 8). Rumors about “the West” also reflected more recent concerns about economic sovereignty in the mineral‐rich region. Since the early 2000s, the DRC has undertaken a process of mining‐sector reform financed by the World Bank, which has provoked a shift from a locally based mining economy to a foreign‐owned model, giving resource sovereignty to transnational corporations and reproducing peripheral marginalization and conflict (Radley, 2023).

In other versions of these rumors, it was neighboring Rwanda that was using the Ebola vaccine to sterilize and exterminate eastern DRC to annex the region, in collaboration with their Western allies. As one trial participant put it: “It is the intention of Rwanda…to take North Kivu and install their population here.” The Ebola vaccine, he explained, was just the first step. In Majengo, a participant took the opportunity to discuss this rumor at length, even though he stressed that he did not believe the rumor to be “true.” Rather, as he explained, it was necessary to situate this rumor in a political context, to understand its significance as a commentary on suffering in the region, and the indifference of Rwanda's Western allies:

The population here has experienced a lot of suffering, during a long period, repetitive war, and this is why we have the rumors saying that the vaccine is poisoned, and that people who administer the vaccine collaborate with Rwanda, because Rwanda didn't succeed in exterminating Congolese by war, they thought it would be better to do it through vaccination.

In the wake of repeated Rwandan military excursions, which have facilitated illegal mineral extraction in North Kivu (Stearns, 2022), these rumors drew on widespread distrust of the neighboring state and experiences of recent violence. Indeed, a year previously, a similar rumor had circulated that the Merck vaccine in the Grand Nord was a Rwandan sterilizing agent (Bisoka et al., 2021).

This rumor focused on malignant Rwanda intentions also draws on a longer standing set of popular idioms and political anxieties. As Muzalia and Rukata (2022, 4) describe, each time the DRC goes through political crisis, “the spectre of ‘balkanization’ arises anew”: the idea that there is a Rwandan conspiracy supported by “the international community,” in particular the USA and UK, to partition DRC, and annex the eastern Kivu region to reconstitute the “Greater Rwanda.” The idea of a “Greater Rwanda” is, itself, a “mythico‐history” (Malkki, 1995), based on the idea that European borders divided a supposedly homogenous group (the Banywarwanda), and that what is now eastern DRC was annexed from precolonial Rwanda (Mathys, 2017). Whilst the historical record is much more complex than this suggests, the notion of a “Greater Rwanda” is used in Rwanda to justify military interference in eastern DRC and remains a dominant frame through which people in DRC understand recurrent military aggressions by Rwanda in the eastern provinces (Mathys, 2017).

Whilst these two rumors focus on the intentions of foreign actors, another rumor described the vaccine as the latest tool of the central Congolese government to weaken the population of a rebellious region, and particularly Nande, who are the majority in Majengo and the Grand Nord. After the failure of the national government to provide security for civilians in the Grand Nord, the start of the Ebola epidemic in the same region, and then the election postponement, many in Majengo concluded that the government was now using another vaccine to target Nande living in Goma. A father from Majengo, Augustin, explained the suspicion among Nande: “See how they [the government] first banned us from voting…And now they are targeting us here with the undesirable consequences of the trial.” Whilst Augustin had volunteered for the trial in the hope that it might protect him from future outbreaks, he had decided to wait to see whether he experienced any negative effects before vaccinating the rest of the family. His own participation was deemed an “acceptable risk,” but Augustin was particularly concerned about the impact of the vaccine on his children and his wife's fertility. Ultimately, the womb came to symbolize the future of the region, where people projected anxieties about possible extermination at the hands of powerful outsiders. Assessments of “acceptable risk” could not be separated from collective political experiences.

## PRAGMATISM AMID ANXIETY

How did this influence pregnant women's experience of the trial? It appeared that men were particularly concerned about potential effects on female fertility; however, biopolitical anxieties and “biopolitical longing” (Geissler, 2012, 217) are not mutually exclusive. Whilst trial participants expressed anxieties, they also hoped for medical protection. Pregnant participants of the trial weighed up the benefits of free healthcare and potential protection, with the uncertainty surrounding potential risks. Far from passive in response to new medical technologies, women were pragmatic and ambivalent (Lock and Kaufert, 1998): they remained simultaneously hopeful about protection and anxious about uncertainty.

Pregnant women described the increasing stigma attached to participating in the trial amid rumors about reproductive health. Yet, many insisted on their ability to make autonomous decisions. Judith, for example, is a trader who lives near the Rwandan border and decided to vaccinate herself and her children. Judith took the first dose of the Ebola vaccine whilst pregnant. The advantages of participating in the trial, she explained, were the possibility of protection from Ebola, and the fact that the costs of the pregnancy were paid for by the trial. Yet, Judith was particularly sensitive to accusations in her neighborhood that she had risked her baby's life for this free healthcare: “There are those who criticized me and asked me if I was not going to be able to give birth. I told them no, even if the maternity is not paid for, I am able and have the right to take this vaccine, nobody is forcing me…I just wanted to be protected by this vaccine like other people.” Ultimately, she stressed that she could choose for herself how she wanted to protect herself and her children.

For other pregnant participants, these rumors became a source of anxiety, leading them to reflect anew on their decision to participate in the trial. Agathe was pregnant when she volunteered for the trial and vaccinated her children. After initially thinking that Ebola was a fiction for elite enrichment, she had been horrified by the impact of the disease whilst living in the Grand Nord. At first, Agathe was hopeful that the trial offered an opportunity for protection that had not been possible with Merck. The policy reversal meant she had access to a potentially lifesaving treatment in an epidemic. After taking the first dose of the vaccine in Goma, however, Agathe became increasingly concerned by the rumors: “When we bring our children to other routine vaccinations, they reassure us with certainty…but for this vaccine, they tell us we have come ourselves and make us sign (a consent form)…we ask ourselves, are we killing ourselves voluntarily?” Rumors do not “only reflect but also contribute to” uncertainty and fear because of the possible truths that they articulate as “narratives of uncertainty” (Samuels, 2015, 234). For Agathe, she felt that she would not know for certain whether the rumors circulating in her neighborhood were “true” until she could see for herself when her baby was born.

It appeared that “trying” and waiting to see what happened became a means to navigate uncertainty. As Susan Whyte (1997) describes, the uptake of new pharmaceuticals is often a pragmatic approach to chronic uncertainty, despite widespread concerns. People navigate uncertainty through action: in Goma, “trying” the vaccine became another pragmatic search for answers. “Personal experiences of evidence” (Butt, 2005)—stories about their own health, or those of fellow participants—then became a source of relief, or anxiety, for pregnant women, as they tried to determine the potential impacts of the vaccine on their bodies. “My mother said that if I go [to be vaccinated], then I will die,” one woman in Majengo described, who was pregnant at the time of taking her first dose. So, “when I arrived at the vaccination center, before they vaccinated me, I asked whether it was true that it [the vaccine] was going to kill people, to sterilize them.” In the end, she decided that “trying” was the only way to find out, and concluded months later that, “it seems to be a good vaccine, well, it hasn't killed us yet!”

The experiences of some pregnant women became “evidence” (Butt, 2005) that challenged rumors about reproductive health. For example, Gertrude is a mother of seven living in Majengo, who described the reaction of her family once she had been vaccinated whilst pregnant: “They [her family] told me that the vaccine will stop my pregnancies because they [the trial] brought it to reduce the population.” Gertrude then went to a neonatal health appointment in the hospital, where people in the waiting room began to laugh at her: “There was a woman who saw my vaccination card and she said ‘oh you had that vaccine? Sorry, your children will be born dead.’” This reaction caused much anxiety for Gertrude. “I was really worried, but then, in the end, I gave birth, and I lived, and my child lived, so then I knew that it was all false,” she concluded.

The experiences of other pregnant women, however, circulated as potential “proof” that the vaccine might have harmful impacts. Another trial participant described having a miscarriage, and her lingering uncertainty about the potential role of the vaccine. The rumors became a way to make sense of her experience: “I was vaccinated during the third month of my pregnancy, and I miscarried in my fourth month. I was so disappointed by this vaccination! When they called me to come and get the second dose, I refused, because I thought that it was what made me miscarry.” However, over time, this participant changed her mind. After extensive conversations with the trial team, she became unsettled in her certainty once again: “the doctors helped me understand that it wasn't the vaccination that led to the miscarriage…I agreed to take the second dose.”

## ETHICAL VERNACULARS

Toward the end of the trial, a man approached me outside one of the clinics. He had already taken both doses of the vaccine but wanted to ask when he would know whether it had “worked”: whether he was protected against Ebola, but also whether the vaccine had any possible side effects, even in the long term. What this man was asking for was an end to his uncertainty—a decisive moment of definitive “proof” that he had not been harmed. Like the pregnant women who had participated, he wanted to understand whether his assessment of “acceptable risk” had, after all, been “acceptable.” Yet, the “results” had not been analyzed by the trial team, nor had they started their lengthy journey toward publication in a scientific journal. This encounter was a stark reminder of how trial participants are so rarely included in the bioethical discussions that affect them, but also the “results” of their participation: conclusions about the relationships between “serious adverse events” and the vaccine, or the “safety outcomes” overall.

Rumors are powerful not just because of their political significance, but because they fill the gaps of contradictory or incomplete information in contexts of uncertainty, becoming a way of “seeking out truth” (Kapferer, 1990, 3). People in Majengo and Kahembe were not part of discussions about eligibility for the Ebola vaccine, nor were they informed of the rationale behind the policy reversal for pregnant women. The decision to include pregnant women did not end ethical debate, nor did it provide certainty. Rather, the reversal opened debate by revealing a degree of scientific uncertainty around notions of “acceptable risk.” Rumors about reproductive health were not simply a reflection of political anxieties, they also acted as a vehicle of ethical debate, or an ethical vernacular. Even for those who did not believe them to be true, sharing rumors, and any potential evidence to support them, became a way of expressing ambivalence about the decision to include pregnant women in the trial, and muddling through uncertainty around the dangers.

Doing rumors, therefore, became a way to fill the gap between the reassurances of the trial team and concerns in the city: a process of sense‐making about contested notions of acceptable risk that are key to the ethics of medical research. Trial participants exchanged experiences of pregnant people they knew in the trial, and the outcomes of these pregnancies. In cases of caesarean section or miscarriage, participants tried to make sense of whether these outcomes could have been influenced by the vaccine. In a focus group in Majengo, for example, a woman described the experience of her sister and friend, who were both participants of the trial and had both needed caesareans. Whilst her friends and family had been quick to attribute this to the vaccine, this participant did not believe the rumors to be true: “I think that it wasn't the vaccine that caused these complications, because after all, there are women who have complications when they haven't received the vaccine.” There were some nods, concerned looks, and questions about whether anyone knew how common caesareans were in the region more generally. “Doing” rumor, therefore, became a way of wrestling with the contentious ethics of medical research in the absence of explanations about why certain decisions had been made, or the “safety results.”

In fact, there was a mirroring between this process of collective meaning‐making among some trial participants and the scientific enquiry amid uncertainty for the trial team. For ethical reasons, there was no unvaccinated control group in the trial, which made the safety results difficult to interpret. Like trial participants, the scientific team turned to a comparison with existing records on pregnancy and neonatal outcomes in eastern DRC to draw further conclusions about the safety of the vaccine on pregnant women. They concluded that further data were needed in this crucial group (Kavunga‐Membo et al., 2024). The experiences of pregnant women in the trial thus became central to the production of two types of “evidence” about the vaccine and its safety amid some uncertainty. For the consortium of global health institutions, the experiences of pregnant women were translated into novel (if imperfect) “data,” critical to grappling with the safety outcomes for this group for the first time. Meanwhile, in Goma, stories about pregnant trial participants circulated as fragments of narrative evidence available in a context of uncertainty. Sharing rumors about the pregnant participants did not constitute “spreading misinformation,” but rather a parallel means of searching for evidence of the effects of the vaccine on reproduction, to make sense of the “acceptability” of risk, and therefore to grapple with the ethics.

## CONCLUSION

The position of pregnant women in clinical research remains a point of contention. During the DRC's 10th Ebola epidemic, decisions about their inclusion or exclusion in vaccine trials were disputed, leading to a policy reversal. This decision to subsequently include pregnant women sparked controversy in Goma, and rumors spread that the vaccine would damage reproductive health. Sterilization has long been an idiom for expressing political anxieties about collective survival in relation to global and national projects. In Goma, such rumors drew on broader concerns about extermination and became public commentaries on recent experiences of foreign extraction, invasion, and state neglect. Faced with this controversy, pregnant women were pragmatic whilst ambivalent, hopeful whilst anxious, when deciding to try experimental therapeutics as a potential source of hope. Rather than simply meaningful political commentaries, however, rumors can also be understood as a form of vernacular ethical debate amid uncertainty. This debate does not simply take place among international bioethical institutions but continues among those most intimately involved in medical research on the ground. Unlike international bioethical debates, ethical vernaculars see biomedical research as inseparable from broader concerns about social justice (Fairhead et al., 2006), foregrounding local political histories and colonial legacies (James et al., 2021). Truly grappling with the ethics of medical research requires serious consideration of how these debates unfold “from below.”

## ENDNOTE

1 Half were women, eight were pregnant.
